# FDX1 serves as a prognostic biomarker and promotes glioma progression by regulating the immune response

**DOI:** 10.18632/aging.204772

**Published:** 2023-06-10

**Authors:** Guangying Zhang, Liangfang Shen, Zhanzhan Li, Yajie Zhao

**Affiliations:** 1 Department of Infectious Diseases, Hunan Key Laboratory of Viral Hepatitis, Xiangya Hospital, Central South University, Changsha 410008, Hunan Province, P.R. China; 2 Department of Oncology, Xiangya Hospital, Central South University, Changsha 410008, Hunan Province, P.R. China; 3 Department of Nuclear Medicine, Xiangya Hospital, Central South University, Changsha 410008, Hunan Province, P.R. China; 4 National Clinical Research Center for Geriatric Disorders, Xiangya Hospital, Central South University, Changsha 410008, Hunan Province, P.R. China

**Keywords:** brainstem glioma, bioinformatics, biomarker, tumor-infiltrating immune cells, prognosis

## Abstract

The present study investigates the prognostic value of the *FDX1* gene and its association with immune infiltration in gliomas. Gene expression profiles and corresponding clinical parameters of glioma patients were obtained from the Cancer Genome Atlas and Chinese Glioma Genome Atlas databases. *In vitro* experiments were also performed to validate its impact on malignant phenotypes of glioma cells. Kaplan-Meier analysis demonstrated that high FDX1 expression was associated with poor prognosis in glioma. Function and pathway enrichment for FDX1 predominantly demonstrated immunomodulatory function. In addition, the high-FDX1 expression group had higher Estimation of Stromal and Immune cells in malignant tumor tissues using Expression data, stromal, and immune scores (*p*<0.001). On evaluation of immunotherapy response, TIDE and dysfunction scores were higher in the low-FDX1 group, while the exclusion score demonstrated an opposite trend. *In vitro* tests showed that FDX1 silencing-induced inhibition of cell invasion and migration inactivated the nucleotide oligomerization domain (NOD)-like receptor signaling pathway by regulating PD-L1 expression. Notably, NOD1 expression was reversed in FDX1-knockdown cells after treatment with NOD1 agonists. In conclusion, FDX1 may play an important role in the diagnosis and treatment of gliomas. Regulating its expression may therefore help improve immunotherapy for these tumors.

## INTRODUCTION

Gliomas are the most common and significant primary central nervous system tumors that demonstrate high invasiveness and mortality [1]. Their prognosis remains dismal despite advancements in standard treatments including neurosurgery, radiotherapy, and chemotherapy [2]. Immunotherapy, which has been developing rapidly in recent years, offers a promising treatment strategy for many cancers. However, owing to the unique tumor immune microenvironment of gliomas, immunotherapy cannot achieve optimal therapeutic effect in many cases [3]. In addition, glioma indicators such as isocitrate dehydrogenase (IDH) 1 and 1p19q do not adequately predict the prognosis in all patients. Hence, exploration of key targets and their relationship with the immune status is of value in the diagnosis of gliomas; it may also help provide precise treatment.

Cumulative research indicates that dysregulation of ferredoxin 1 (FDX1) is involved in the development and progression of various tumors [4]. The *FDX1* gene encodes an acidic iron-sulfur protein (14 kDa), which is located in the mitochondrial matrix; it is involved in many metabolic processes including steroidogenesis, bile acid synthesis, iron-sulfur cluster biogenesis, and vitamin D synthesis [5, 6]. Evidence suggests that aberrant metabolism is closely linked to the immune microenvironment during cancer development [7]. FDX1 possibly transfers electrons to mitochondrial cytochrome P450 enzymes allowing them to perform the catalytic reactions. In this context, researchers have found that modification of FDX1 concentrations affects its catalytic activity *in vitro* [8]. A novel type of copper-induced cell death (cuproptosis) has recently been discovered; it differs from the other modes of cell death including apoptosis, pyroptosis, necroptosis, and ferroptosis [9–11]. Studies indicate that FDX1 is a critical mediator of cuproptosis. However, its role in the prognostication of gliomas is relatively under-studied. It is therefore necessary to determine its function and immune role in these tumors.

In the present study, we investigated the expression and prognostic value of the *FDX1* gene in gliomas by performing database analyses and constructing Kaplan-Meier curves. Gene Ontology (GO) and Kyoto Encyclopedia of Genes and Genomes (KEGG) analyses were performed to evaluate the biological functions of FDX1. We also studied the relationship between FDX1 and immune related scores in glioma patients to explore potential immunotherapy options. We additionally determined the expression of FDX1; the findings suggested that FDX1 could promote the invasion and migration ability of glioma cells *in vitro*. The findings also indicated that FDX1 may serve as a survival indicator and potential therapeutic target in gliomas. The results provided valuable insight into the mechanisms involved in the association between FDX1 and cancer-immune interactions in glioma.

## MATERIALS AND METHODS

### Data sources

Gene expression profiles, mutation data, and clinicopathological parameters of glioma patients were acquired from The Cancer Genome Atlas (TCGA, https://portal.gdc.cancer.gov/) and Chinese Glioma Genome Atlas (CGGA, http://www.cgga.org.cn/) databases. All data pertaining to clinical factors, including age, gender, World Health Organization (WHO) stage, histology, tumor type, 1p19q codeletion status, receipt of radiotherapy and chemotherapy, IDH mutation status, and survival status were downloaded for further analysis. Data regarding immune cell infiltration levels and immune-related function were obtained from the Cancer Imaging Archive (https://tcia.at/home) database.

### Bioinformatic analysis

Differentially expressed genes (DEGs) were detected based on clinical parameters using the R software package. Patients were divided into high- and low-FDX1 expression subgroups based on the median FDX1 expression level; function and pathway enrichments were then investigated in the two groups. Gene Set Variation Analysis (GSVA) was used to estimate the enrichment status of biological processes. GO and KEGG analyses were also performed using the cluster Profiler R package to explore the function of *FDX1* in biological processes. Univariate and multivariate Cox regression was performed using data from the TCGA and CGGA datasets to validate the independence of the risk score (messenger ribonucleic acid [RNA] expression multiple divided by the FDX1 regression coefficient); the survival package of R was used for analysis. The hazard ratio (HR), 95% confidence intervals (CI), and log rank p-values were also evaluated. Stratified analysis of different clinical characteristics was performed to evaluate the association between FDX1 expression and survival data in patients with glioma; the characteristics included age, gender, WHO stage, histology, primary-recurrent-secondary (PRS) type, codeletion status, and receipt of radiotherapy and chemotherapy. Kaplan-Meier survival analysis was performed to compare patient prognosis between the high- and low- FDX1 expression groups. Tumor Immune Estimation Resource (TIMER) was used to evaluate the correlation of FDX1 expression with immune cell infiltration and immune checkpoint status. The proportions of 16 immune cell infiltration levels and immune-related function scores were also analyzed for each sample from the high- and low- FDX1 expression groups [12]. Pearson correlation analysis was employed to estimate the association between FDX1 expression levels and macrophage (M0, M1, and M2), monocyte, activated CD4 memory T cell, CD8 T cell, gamma delta T cell, activated natural killer (NK) cell, and resting CD4 memory T cell counts; *p* values of < 0.05 were considered to indicate significant correlation. Immunotherapy response was predicted using the Tumor Immune Dysfunction and Exclusion (TIDE) algorithm [13, 14].

### Cell culture and transfection

U251, U87, and normal human astrocyte cell lines were acquired from Procell (Wuhan, China). The cell lines were cultured in Dulbecco’s modified Eagle’s medium supplemented with 10% fetal bovine serum (Gibco, USA) and grown in an incubator at 37° C in 5% CO_2_. The lentiviral vector (GV493) carrying short hairpin RNA (shRNA) of FDX1 was constructed by Genechem (Shanghai, China). The sense primer was 5′- TTCAACCTGTCACCTCATCTTTG -3′ and the antisense primer was 5′- TGCCAGATCGAGCATGTCATT -3′. For NOD1, the plasmid was obtained from Transheep (Shanghai, China). pGL6-TA-luc-NOD1, control vector, shRNA, and non-specific shRNA control were transfected into U87 and U251 cells with Lipofectamine 3000 (Invitrogen, USA) as per the manufacturer’s protocol. They were screened with puromycin (0.5 mg/L, Sigma-Aldrich) after 10 days and U87 and U251 stable cells were generated; these cells expressed FDX1- shRNA.

### Quantitative real-time-polymerase chain reaction

Total RNA was extracted using Trizol (Invitrogen, USA) and complementary deoxyribonucleic acid was synthesized using the PrimeScriptTM RT Reagent Kit (TaKaRa, Japan) according to the manufacturer’s instructions. The primer sequences for FDX1 were obtained from Santa-Cruz Biotechnology. Quantitative real-time polymerase chain reaction (qRT-PCR) was performed using the SYBR premix Taq kit. The relative expression of FDX1 was normalized to that of glyceraldehyde 3-phosphate dehydrogenase and calculated by the 2 ^−ΔΔCt^ method. The primer sequences of SRY-box transcription factor 2 (SOX2), matrix metallopeptidase 9 (MMP9), vimentin, programmed cell death 1 (PDL1), and nucleotide binding oligomerization domain containing 1 (NOD1) are provided in Supplementary Table 1.

### Western blot

The cells were lysed with radioimmunoprecipitation lysis buffer (Beyotime, China). Proteins were then separated by sodium dodecyl-sulfate polyacrylamide gel electrophoresis and transferred on to polyvinylidene fluoride membranes (Millipore, USA). After blocking the membranes with 5% non-fat milk for 1 h, they were washed thrice using Tris Buffered Saline with Tween (5 min each). The membranes were then incubated overnight with primary antibody (1:1000 dilution) at 4° C on a shaking bed. Horseradish peroxidase-conjugated secondary antibody (Thermo Fisher Scientific, USA) was added the next day and the membranes were incubated for 1.5 h. After washing with Tris Buffered Saline with Tween, the protein was visualized using horseradish peroxidase enhanced chemiluminescence substrate (Advansta, USA) and the results were photographed by the imaging system (Bio-Rad, USA). Specific primary antibodies against PD-L1 (Cell Signaling Technology, USA), FDX1 (Biorbyt, UK), and NOD1 (Cell Signaling Technology, USA) were used; β-tubulin was used as the internal reference.

### Transwell assay

Cell migration and invasion assays were plated in 24-well transwell chambers (with or without Matrigel coating on the upper surface of the membrane) (Corning, USA) to detect migration and invasion ability. The upper chamber was filled with serum free medium and the lower one was immersed in complete media containing 10% fetal bovine serum. At 24 hours after incubation, the migrated and filtered invasive cells were fixed with paraformaldehyde and colored with crystal violet. Migrated and invaded cells were then counted and photographed using a light microscope (Olympus, Japan) in 5 random fields for each chamber; 3 experiments were performed in triplicate.

### Scratch assay

U87 and U251 cells were seeded in 6-well plates at a density of 5 × 10^5^cells /well and incubated at 37° C with 5% CO_2_. Straight scratches were placed on monolayer cells with a pipette tip and floating cells were washed off using phosphate-buffered saline; glioma cells were cultured in serum-free medium. Finally, the distance between two edges of a scratch were measured under a light microscope (Leica, Germany) to calculate the migration ability of each group of cells. The above steps were repeated thrice.

### Statistical analysis

The limma R package was used to analyze DEGs. The chi-square test was used to compare the differences between category variables. One-way analysis of variance was used to assess the difference in *FDX1* gene expression among all grades of gliomas and non-tumor lesions. We performed the log-rank test to evaluate the overall survival (OS) curves of the high- and low- FDX1 groups; univariate and multivariate Cox regression was performed and the corresponding HR with 95% CIs were also calculated. All statistical analyses were conducted using R version 4.0.1; differences with *p* values of < 0.05 were considered significant.

### Data availability

The data can be partly available from the public database (TCGA and CGGA). Data from experiments can be available from the corresponding authors upon request.

## RESULTS

### General information regarding FDX1

**Figure 1:**
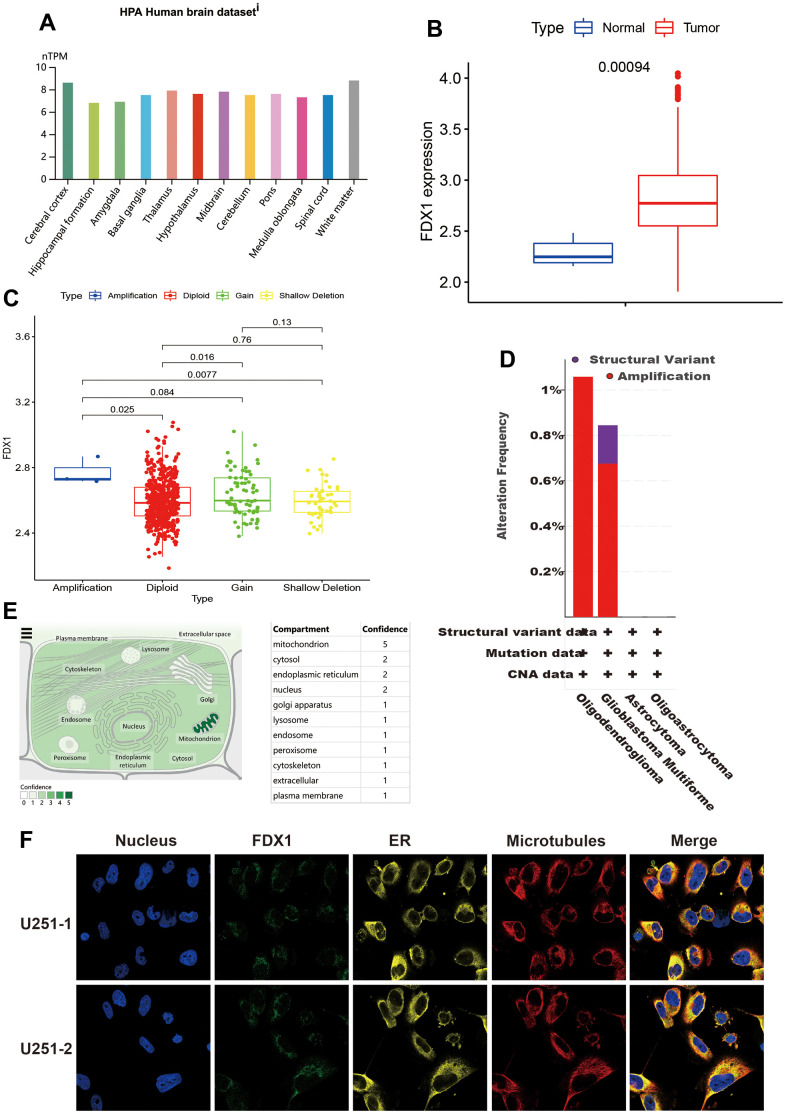
**General information regarding FDX1.** (**A**) Expression distribution of FDX1 in brain tissue. (**B**) FDX1 expression is higher in the tumor tissue than in normal tissue. (**C**) Comparison of FDX1 expression among different copy number variations. (**D**) FDX1 alteration frequency by glioma subtype. (**E**) Subcellular location of FDX1. (**F**) Immunofluorescence images of FDX1 protein, nucleus, endoplasmic reticulum, microtubules, and merged images in U251 cells.

We first analyzed FDX1 expression in human brain tissue. As shown in Figure 1A, FDX1 was significantly overexpressed in various brain locations, including the cerebral cortex, white matter, thalamus, midbrain, pons, basal ganglia, spinal cord, medulla oblongata, cerebellum, hypothalamus, amygdala, and hippocampal formation. As presented in Figure 1B, *FDX1* was upregulated in tumor tissue as opposed to normal brain tissue (*p* <0.001). To explore the roles of FDX1 in glioma, we evaluated the correlation between FDX1 expression and copy number variations; we found that copy number variations mainly included diploidy, gain and shallow deletions, and small amplification ratios. The expression of FDX1 was increased in cases of amplification than in diploidy and shallow deletion (*p* <0.05). In addition, FDX1 was more prone to having gain mutations than diploid mutations (all *p*<0.05, shown in Figure 1C). Two or more alterations of *FDX1* were detected in different subtypes of glioma; notably, amplification alterations were more common in glioma samples (Figure 1D). The subcellular localization of FDX1 was identified for better understanding of its function; FDX1 was mainly found in the mitochondria, cytosol, endoplasmic reticulum, and nucleus (Figure 1E). As seen in the overlaid image, there were at least partial colocalizations of FDX1 in both the endoplasmic reticulum and microtubules in U251 cells; as illustrated by Figure 1F, all three were present at the cell periphery.

### FDX1 and prognosis in glioma patients

**Figure 2:**
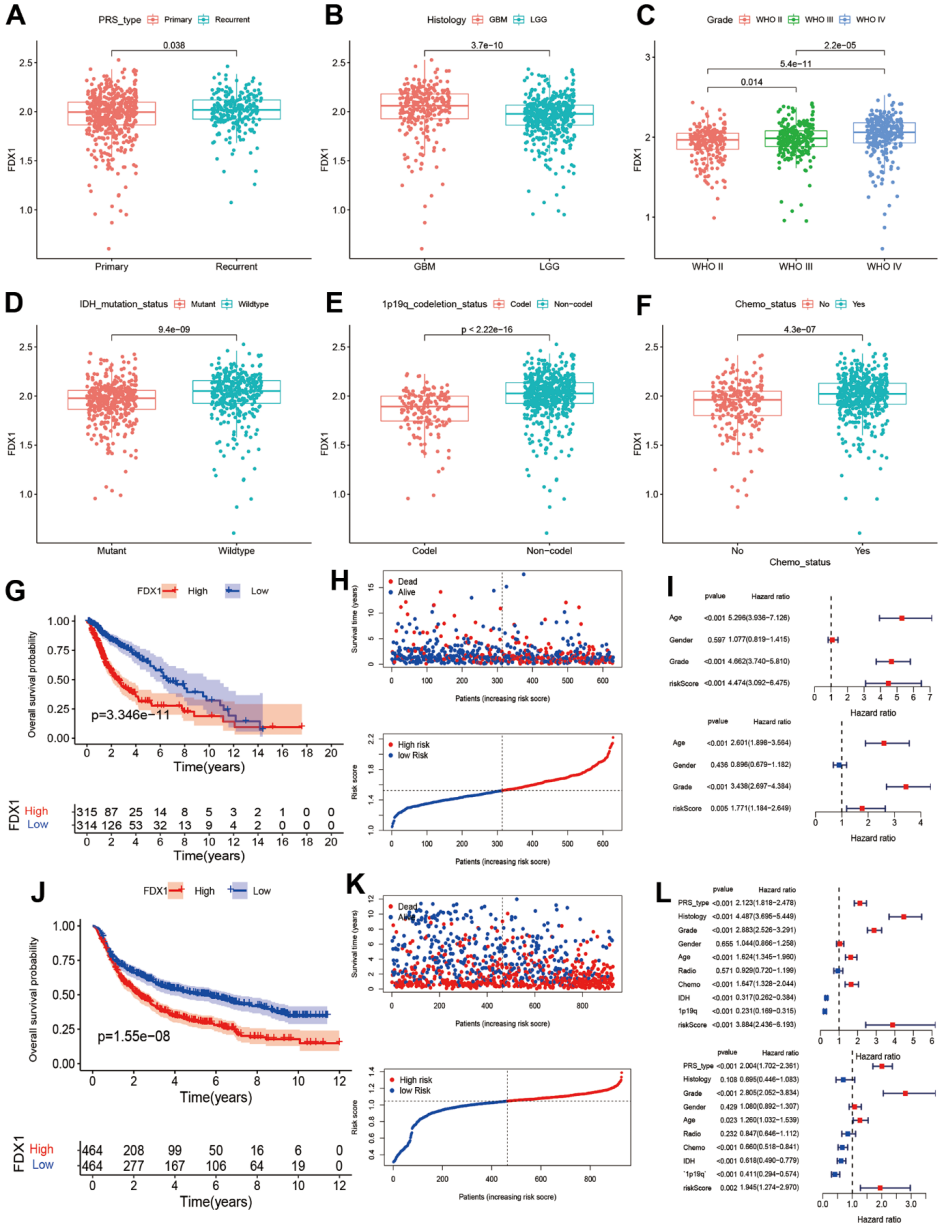
**Association between FDX1 and prognosis in glioma.** (**A**–**F**) Comparison of FDX1 expression levels based on different tumor types, histology, grade, IDH mutation status, 1p19q codeletion status, and chemotherapy status. (**G**, **H**) Kaplan-Meier curves from TCGA data indicate that high FDX1 expression is associated with poor prognosis. (**I**) Univariate and multivariate Cox regression performed using TCGA data show FDX1 to be an independent prognostic factor in glioma. (**J**, **K**) Kaplan-Meier curves from CGGA data show high FDX1 expression to be associated with poor prognosis. (**L**) Univariate and multivariate Cox regression using CGGA data indicate FDX1 to be an independent prognostic factor in glioma.

We investigated the correlation between FDX1 and clinicopathological parameters of glioma. As shown in Figure 2A–2F, FDX1 expression was increased in cases with tumor recurrence, advanced grade, wild type IDH, 1p19q non-codeletion, and history of chemotherapy administration (*p* <0.05). The above results demonstrate that FDX1 expression is associated with these clinicopathological characteristics of gliomas. The Kaplan-Meier curves showed that the groups with high FDX1 expression in TCGA and CGGA had a poor prognosis (Figure 2G, 2J). We then analyzed the survival status distribution based on FDX1 expression. The results indicated that survival times and rates were significantly decreased in patients with high FDX1 expression compared with those having low expression (Figure 2H, 2K). Univariate Cox regression analysis showed that age (HR=5.296, *p* < 0.001), grade (HR=4.662, *p* < 0.001) and risk score (HR=4.474, *p* < 0.001) were significantly associated with OS in the TCGA cohort. Multivariate Cox regression indicated that FDX1 expression (HR=1.771, *p*=0.005) was an independent prognostic factor for glioma in the TCGA cohort (Figure 2I). In the CGGA cohort, univariate Cox regression analysis showed that PRS type (HR=2.123, *p* < 0.001), histology (HR=4.487, *p* < 0.001), grade (HR=2.883, *p* < 0.001), age (HR=1.624, *p* < 0.001), receipt of chemotherapy (HR=1.647, *p* < 0.001), IDH mutation status (HR=0.317, *p* < 0.001), 1p19q codeletion status (HR=0.231, *p* < 0.001), and risk scores (HR=3.884, *p* < 0.001) were associated with OS. Multivariate Cox regression showed that FDX1 expression (HR=1.945, *p*=0.002) was an independent prognostic indicator for glioma (Figure 2L); the CGGA provided similar findings. A nomogram was also constructed to predict patient survival times by combining FDX1 expression and clinicopathological indicators including FDX1 expression, age, chemotherapy status, IDH mutation status, 1p19q codeletion status, PRS type, and grade. Finally, we calculated the total score based on the points assigned for each parameter (Supplementary Figure 1).

### Clinical characteristics with different expression levels of FDX1

**Figure 3:**
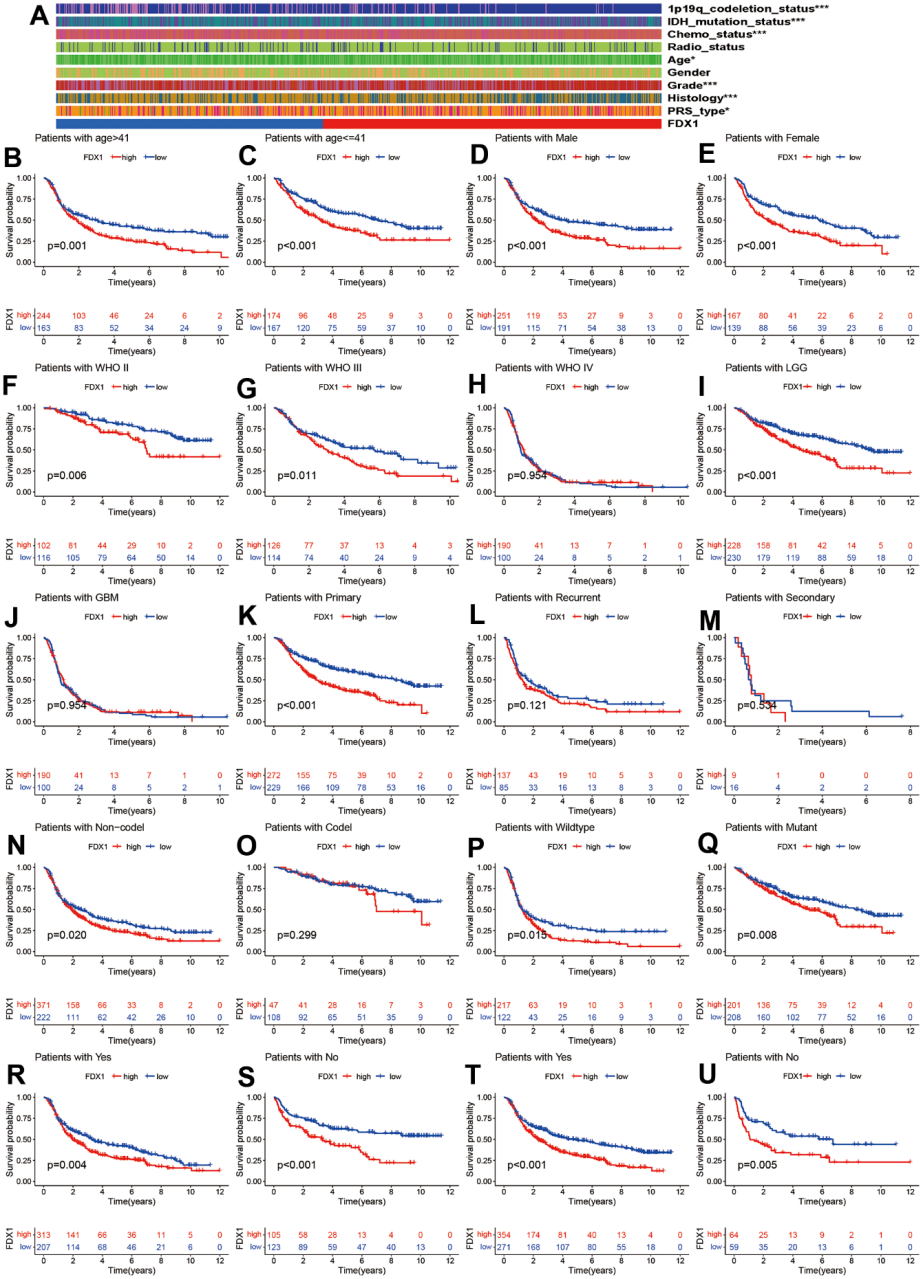
**Correlation of FDX1 with clinical characteristics.** (**A**) Clinical characteristics in high- and low-FDX1 expression groups. (**B**–**U**) Stratified analysis of correlation of FDX1 with prognosis in different groups: age (> 41 vs. ≤41), gender (male vs. female), WHO stage (II, III, and IV), histology (LGG vs*.* GBM), tumor type (primary, recurrent, and secondary), 1p19q codeletion status (non-codeletion vs. codeletion), IDH mutation status (wildtype vs. mutant), and receipt of radiotherapy and chemotherapy (yes vs. no).

We compared the distribution of clinical characteristics between the high- and low-FDX1 expression groups. As seen in Figure 3A, the red and blue bars represent the high and low FDX1 groups, respectively. The heatmap indicated that 1p19q codeletion, IDH mutation status, chemotherapy, age, tumor grade, histology, and PRS type differed significantly between the high- and low-FDX1 groups (*p* < 0.05). As shown in Figure 3B–3U, we had also performed stratified analysis by different clinical factors in the high-and low-FDX1 groups. Kaplan Meier analysis demonstrated the OS rates in different FDX1 expression groups stratified by age (> 41 y vs. ≤ 41 y), gender (male vs. female), glioma grade (II, III, and IV), histology (low grade glioma [LGG] vs. glioblastoma multiforme [GBM]), tumor type (primary, recurrent, and secondary), 1p19q codeletion status (non-codeletion vs. codeletion), IDH mutation status (wildtype vs. mutant), and receipt of radiotherapy and chemotherapy (yes vs. no). The results showed that the OS was shorter in the high FDX1 expression group than in the low FDX1 expression group in the following cases: age > 41 (*p*=0.001), age ≤ 41 (*p* <0.001), male (*p* <0.001), female (*p* <0.001), grade II (*p* =0.006), grade III (*p*=0.011), LGG (*p* <0.001), primary tumor (*p* <0.001), 1p19q non-codeletion (*p*=0.020), IDH wildtype (*p*=0.015), IDH mutations (*p*=0.008), receipt of radiotherapy and chemotherapy (*p*=0.004 and *p* <0.001), and non-receipt of radiotherapy and chemotherapy (*p* <0.001 and *p*=0.005).

However, the OS rate was statistically similar in cases of grade IV glioma, GBM, recurrent and secondary tumors, and 1p19q non-codeletion. Patients with glioma had a poor prognosis irrespective of high- or low-FDX1 expression; this was probably related to the smaller sample size or the individual clinical parameters.

### Function and pathway enrichment for FDX1

**Figure 4:**
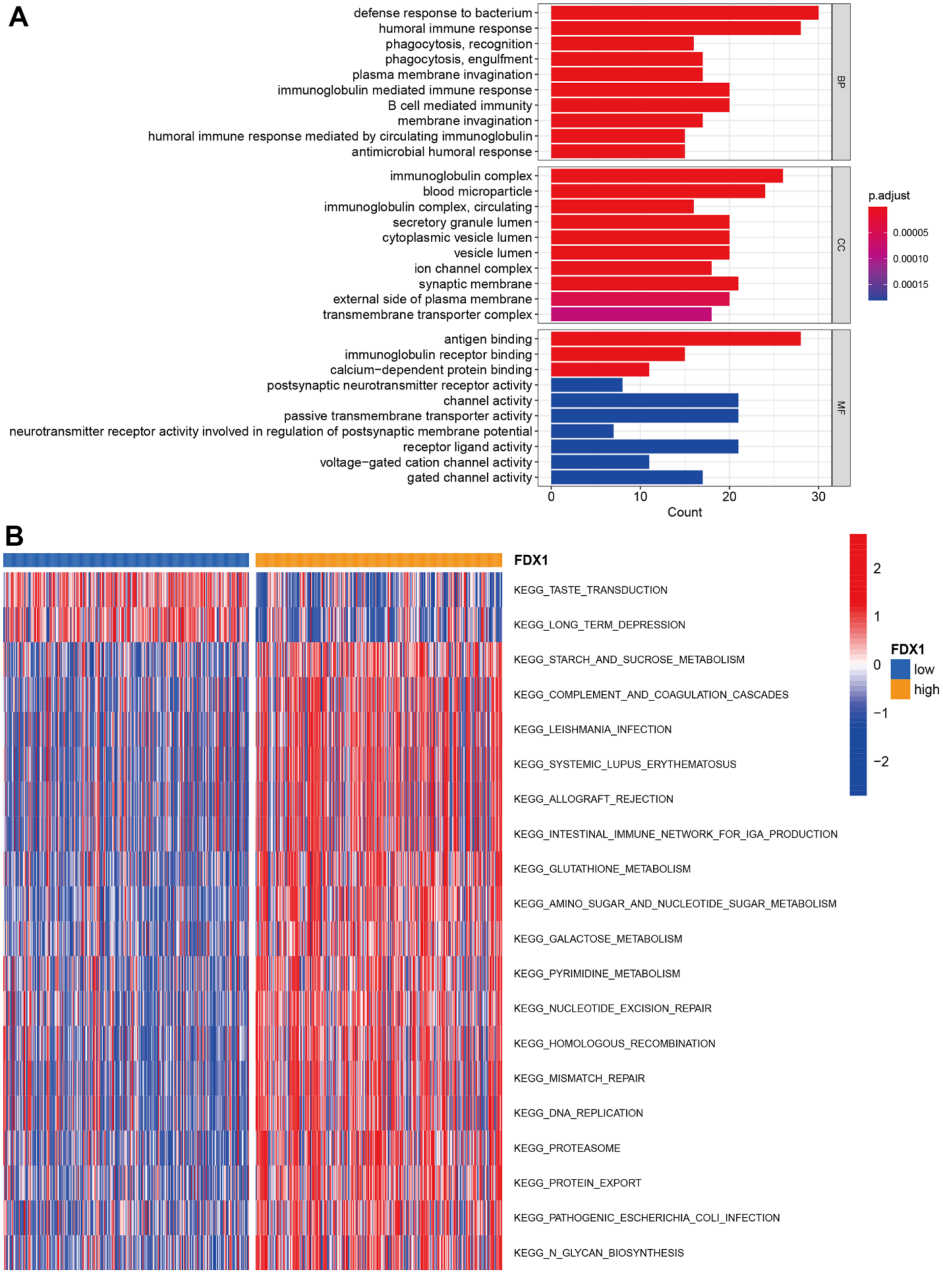
**Function and pathway enrichment analysis.** (**A**) GO enrichment analysis. (**B**) GSVA analysis in high- and low-FDX1 expression groups.

We performed GO enrichment analysis to investigate the potential biological differences between the high- and low-FDX1 expression groups. The top three terms during GO analysis for biological processes were defense response to bacteria, humoral immune response, and immunoglobulin-mediated immune response; for the cellular component, these mainly involved immunoglobulin complexes, blood microparticles, and synaptic membranes. Molecular function analysis indicated abundance of DEGs in antigen binding (Figure 4A). GSVA analysis showed significant differences in enrichment between the two groups in terms of taste transduction and long-term depression, among others (Figure 4B). KEGG analysis suggested that DEGs were enriched in neuroactive ligand-receptor interaction; the details are presented in Supplementary Figure 2. The above function and pathway enrichment analysis results indicated that most enriched pathways manifested immunomodulatory functions.

### Correlations of FDX1 with the tumor microenvironment and immune infiltration

**Figure 5:**
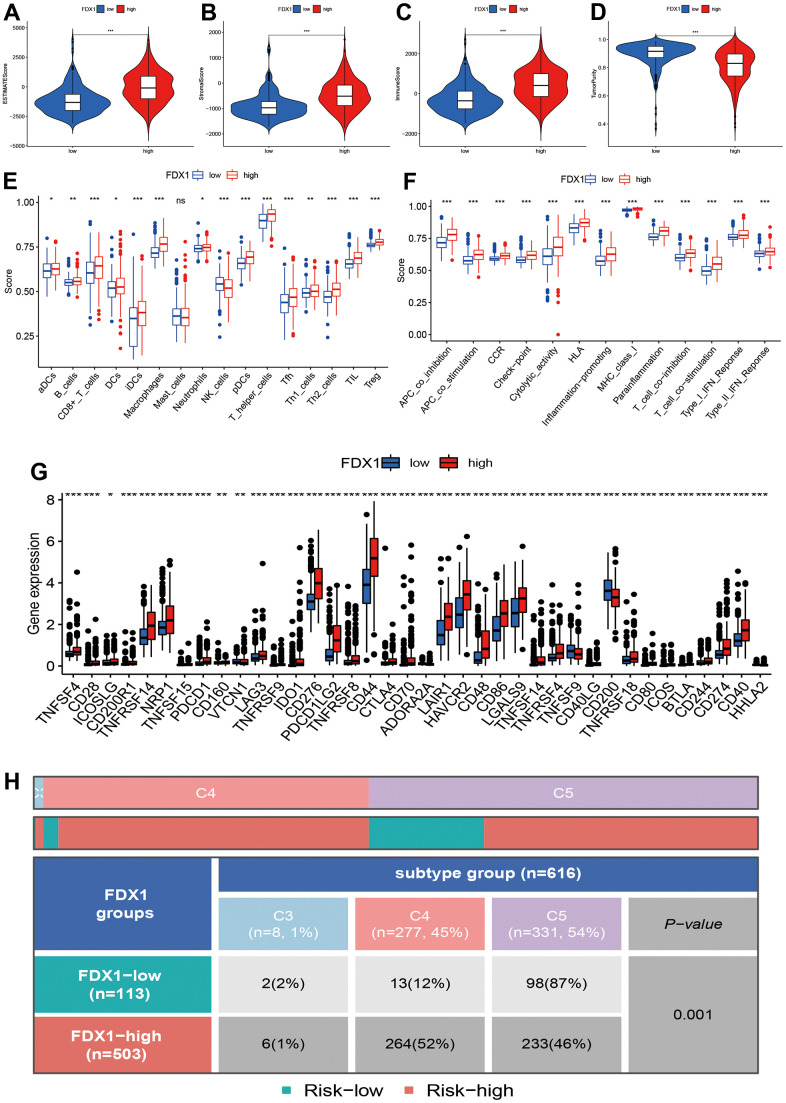
**Correlation of FDX1 expression with immune status.** (**A**–**D**) Association between FDX1 expression and ESTIMATE, stromal, and immune scores and tumor purity. (**E**, **F**) Immune cell infiltration levels and immune-related function in high- and low-FDX1 expression groups. (**G**) Differences in expression of immune checkpoint genes between high- and low- FDX1 expression groups. (**H**) Correlation between FDX1 and immune subtypes.

In order to explore the correlations of FDX1 expression with immune status and immunotherapy, we first calculated the Estimation of STromal and Immune cells in MAlignant Tumor tissues using Expression data (ESTIMATE) score, stromal score, immune score, and tumor purity in different FDX1 expression groups. The high-FDX1 expression group had higher ESTIMATE, stromal, and immune scores; however, the distribution of tumor purity demonstrated an opposite trend (Figure 5A–5D, *p*< 0.001). We further compared the abundance of infiltration by 16 immune cells in the 2 groups. As shown in Figure 5E, the proportions of all immune cells (except for mast cells) were increased in the high-FDX1 expression group. Immune functions such as human leukocyte antigen-mediated regulation, inflammation-promotion, and antigen presenting cell co-inhibition were all increased in the high-FDX1 expression group (Figure 5F). The results indicate that FDX1 plays an important role in the cancer immune microenvironment.

**Figure 6:**
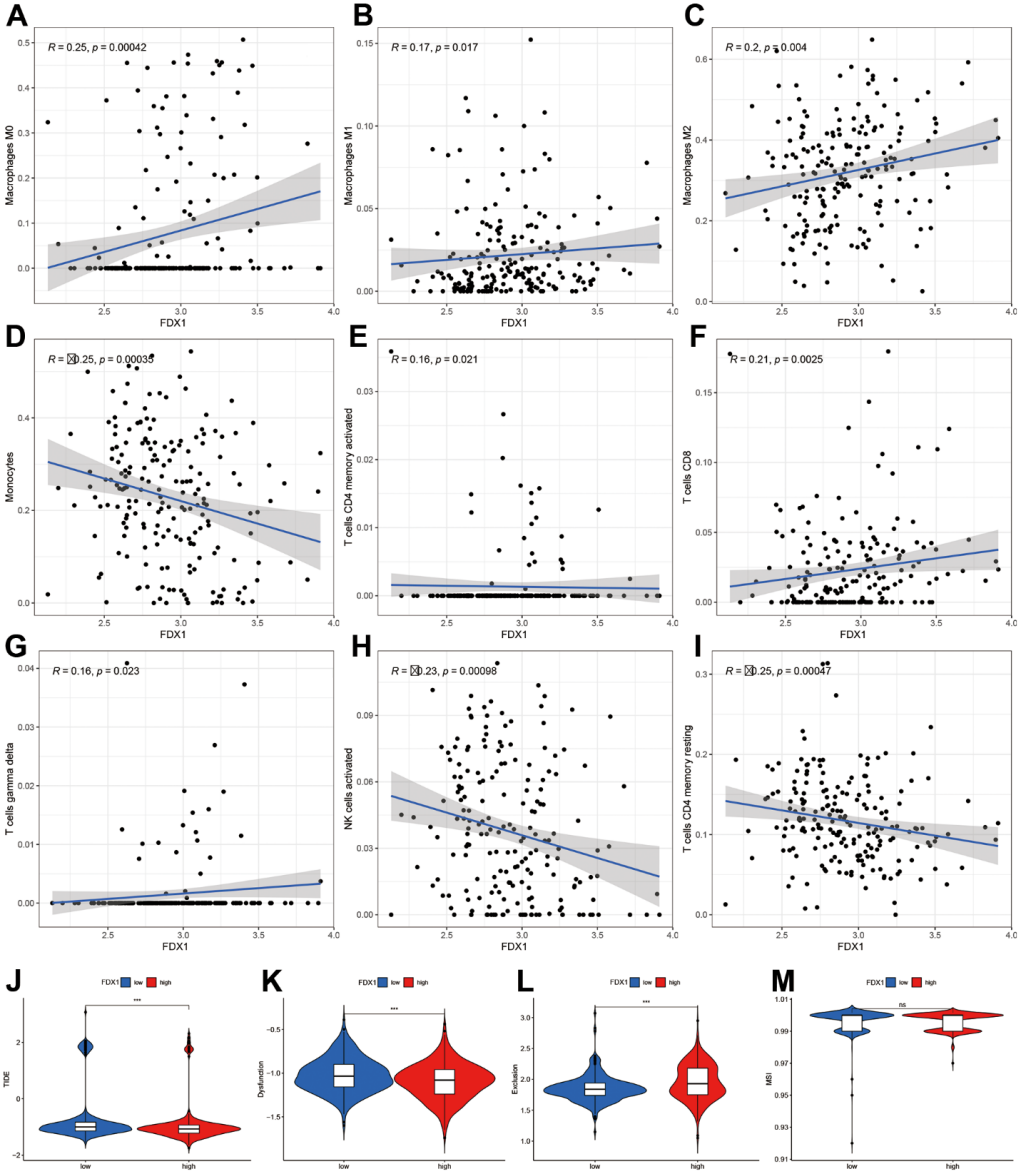
**Correlation of FDX1 expression with immune cells and immunotherapy.** (**A**–**I**) Scatter plot indicating the association between FDX1 and macrophages (M0, M1, and M2), monocytes, activated CD4 memory T cells, CD8 T cells, gamma delta T cells, activated NK cells, and resting CD4 memory T cells. (**J**–**M**) Correlation between FDX1 and immunotherapy response using TIDE, dysfunction, exclusion, and MSI.

A high infiltration of immune cells was also observed in patients with high-FDX1 expression. We therefore speculated that the cancer cells in the high-FDX1 expression group may have had upregulated the immune-suppressive checkpoints to evade attack by immune cells. We then compared the expression levels of immune checkpoints in the different groups. The results revealed that the expression of most immune checkpoint genes was remarkably higher in the high-FDX1 expression group compared with the low expression group (Figure 5G); the differences in immune subtypes among the high- and low-FDX1 expression groups are presented in Figure 5H. The high-FDX1 expression group mainly included the C5 subtype and the low-FDX1 expression group mainly included the C4 and C5 subtypes. However, the proportion of C3 subtype cases was less in both groups. The scatter plot indicated that FDX1 expression correlated positively with macrophage (M0, M1, and M2), activated CD4 memory T cell, CD8 T cell, and gamma delta T cell counts. However, monocytes, activated NK cells, and resting CD4 memory T cells were downregulated with increased FDX1 expression (Figure 6A–6I); the stratified analyses indicated a similar trend (Supplementary Figure 3).

Finally, we analyzed the TIDE, dysfunction, exclusion, and microsatellite instability (MSI) scores to examine the immunotherapy response of patients with glioma. The results indicated that the TIDE and dysfunction scores were lower in the high-FDX1 expression group than in the low expression group, while the exclusion score demonstrated an opposite trend. There was no significant difference in MSI scores between the two groups (Figure 6J–6M). In conjunction, these findings demonstrated differences in immune infiltration and immunotherapy responses between high- and low-FDX1 expression groups. The findings also suggested that tumors with high-FDX1 expression could be susceptible to immunotherapies due to the involvement of many immune targets.

### FDX1 promotes glioma progression by regulating immune surveillance via NOD-like receptor signaling pathway activation

**Figure 7:**
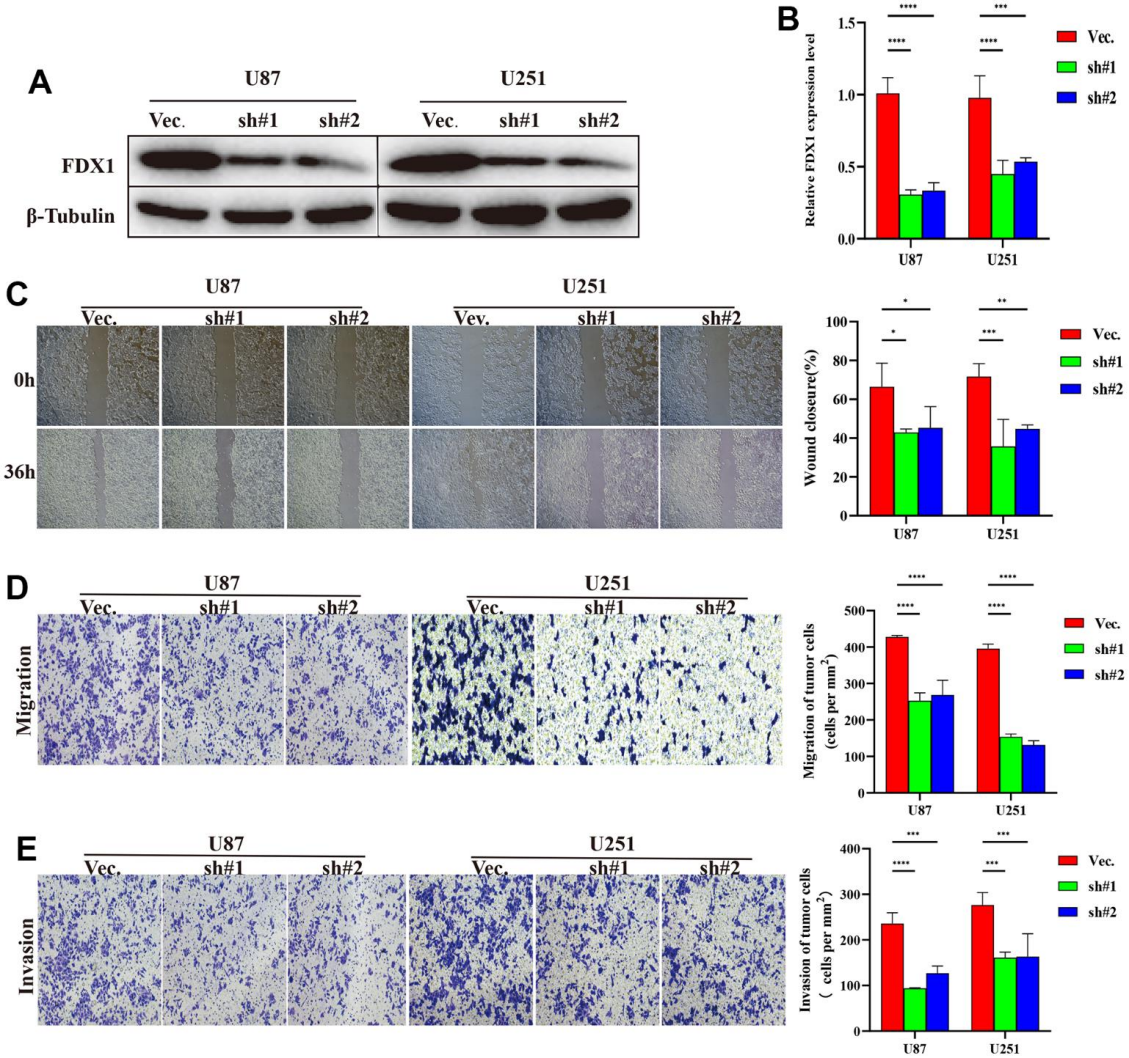
**FDX1 silencing inhibits the progression of glioma.** (**A**) Western blot of FDX1 expression in endogenous FDX1-knockdown U87 and U251 cell lines. (**B**) qRT-PCR indicating FDX1 knockout in U87 and U251 cell lines. (**C**) Scratch assay showing that FDX1 silencing inhibits migration ability. (**D**, **E**) Transwell assay indicating that FDX1 silencing suppresses migration and invasion ability in glioma cells.

**Figure 8:**
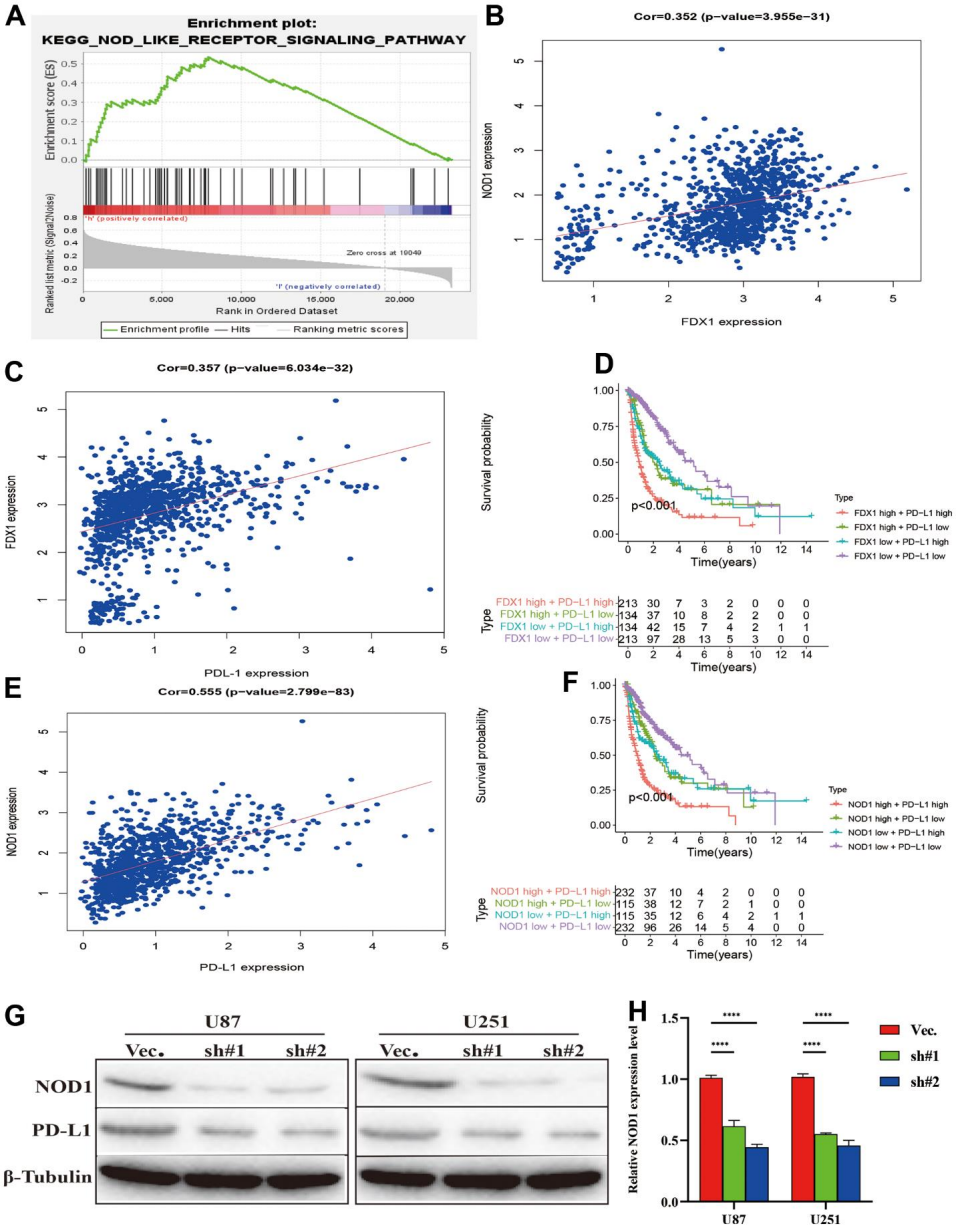
**Silencing FDX1 inhibits PD-L1 expression.** (**A**) GSEA indicates that the NOD-like receptor signaling pathway is positively enriched with high FDX1 expression. (**B**, **C**) FDX1 expression is positively associated with NOD1 and PD-L1. (**D**) FDX1 and PDL1 co-expression confers poorest survival outcomes. (**E**) NOD1 expression is positively associated with PD-L1. (**F**) NOD1 and PDL1 co-expression confers the poorest survival outcomes. (**G**) Western blot indicates that NOD1 and PD-L1 are inhibited after FDX1 silencing. (**H**) qRT-PCR shows low NOD1 expression after FDX1 silencing.

**Figure 9:**
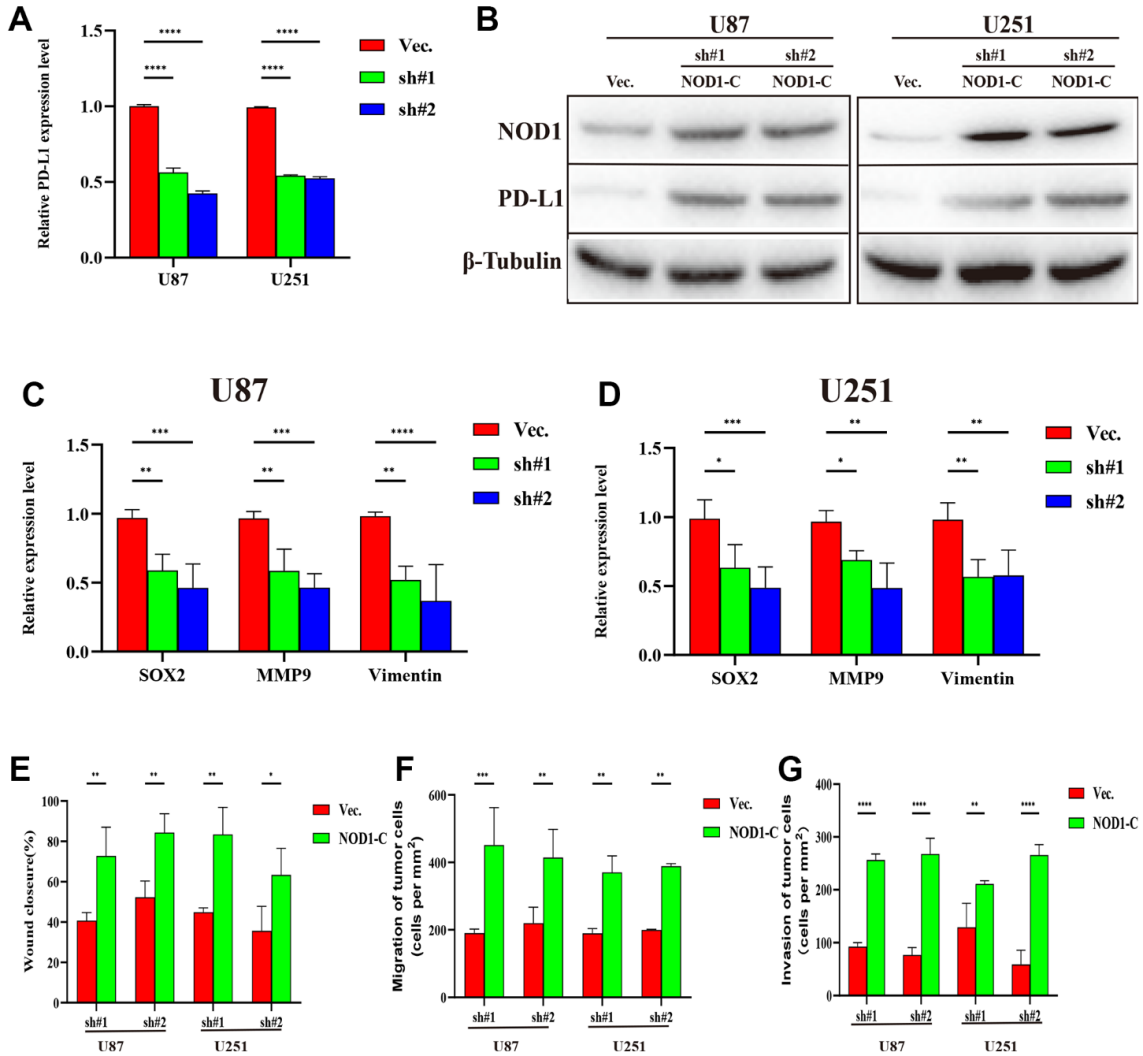
**Silencing FDX1 inhibits glioma progression via the NOD1-PDL1 axis.** (**A**) qRT-PCR shows low PDL1 expression after FDX1 silencing. (**B**) NOD1 and PDL1 levels are upregulated in FDX1-silenced cells after NOD1-C treatment. (**C**, **D**) Key molecules of migration decreased after FDX1 silencing. (**E**) Wound closure ratio elevated in FDX1-silenced cells. (**F**, **G**) Number of migrating and invading tumor cells increased in FDX1-silenced cells after NOD1-C treatment.

We performed experiments *in vitro* to explore the FDX1-mediated mechanisms in glioma progression and validate the effect of FDX1. We also constructed FDX1-knockdown glioma cell lines (U87 and U251, Figure 7A, 7B). The scratch assay indicated a decrease in the migration ability of glioma cells after FDX1 silencing (Figure 7C). The transwell assay further showed that FDX1 silencing inhibited the invasion and migration ability of glioma cells (Figure 7D, 7E). We performed KEGG enrichment analysis to explore the mechanism of FDX1-mediated regulation in glioma progression; we found that the NOD-like receptor signaling pathway was positively enriched in the high FDX1 expression group (Figure 8A). FDX1 was also positively associated with *NOD1*, a key gene of the NOD-like receptor signaling pathway (Figure 8B). Previous studies have suggested that the NOD-like receptor signaling pathway plays an important role in the innate immune response. We further explored the correlation between FDX1 and immune checkpoint gene *PD-L1*; a positive association was observed (Figure 8C). The group with FDX1 and PD-L1 co-expression had the worst prognosis (Figure 8D). NOD1 also showed positive association with PD-L1 (Figure 8E); the group with co-expression of NOD1 and PD-L1 also had the poorest prognosis (Figure 8F). The Western blot and quantitative PCR results indicated a decrease in NOD1 and PD-L1 after FDX1 silencing (Figures 8G, 8H, 9A). These results show that FDX1 silencing-induced inhibition of cell invasion and migration is associated with inactivation of the NOD-like receptor signaling pathway.

We further examined the effect of NOD-like receptor signaling pathway activation on FDX1-mediated cell invasion and migration in U87 and U251 cells. The Western blot indicated that NOD1 levels were reversed in FDX1-silenced cells after NOD1-C treatment (Figure 9B). The quantitative PCR results showed that the expression levels of the migration markers (SOX2, MMP9, and Vimentin) had decreased significantly in FDX1-silenced cells compared with those in the vector group (Figure 9C, 9D). The scratch and transwell assays showed that active NOD1 in FDX1-silenced glioma cells increased invasion and migration ability (Figure 9E–9G). We further examined the methylation level of FDX1 in glioma and found that the promoter of FDX1 showed low methylation levels. The methylation level decreased with an increase in tumor grade, with WHO grade IV tumors demonstrating the lowest methylation level; low methylation of FDX1 was also associated with a short progression free interval (Supplementary Figure 4). In addition, low methylation of FDX1 promoter increased the expression of FDX1 messenger RNA. These results indicate that FDX1 may promote the progression of gliomas by regulating PD-L1 expression (via activation of the NOD-like receptor signaling pathway).

## DISCUSSION

Our study found that FDX1 levels were highly elevated in glioma tissue. High levels of FDX1 expression were found to be associated with poor OS. Clinical characteristics and biological function were found to differ between high and low FDX1 expression groups. Multivariate Cox regression indicated that FDX1 was an independent prognostic factor for patients with glioma. Tumor microenvironment and immune cell infiltration levels were found to differ significantly between groups with high and low FDX1 expression levels. The experimental results indicated that FDX1 silencing-induced inhibitions of cell invasion and migration were associated with inactivation of the NOD-like receptor signaling pathway (via regulation of PD-L1 expression); this provided valuable insights into the role of FDX1 in the progression of glioma.

Gliomas are one of the most lethal brain tumors with a poor prognosis. The average survival rate of decreases gradually with increase of WHO grade. Although optimization of traditional therapeutic regimens can improve clinical outcomes, no breakthroughs have been achieved in the treatment of glioma over the past decade [15, 16]. There is therefore an urgent need for the identification of new prognostic indicators and therapeutic approaches. Various specific molecular alterations have recently been identified in gliomas; these include 1p/19q codeletion, IDH1 mutations, and O6-methylguanine-deoxyribonucleic acid methyltransferase promoter methylation. These alterations have been determined to be predictive factors and therapeutic targets [17, 18]. The development of immunotherapy has led to the use of active or passive immunotherapies and immune checkpoint inhibitor therapies in gliomas [19, 20]. However, glioma heterogeneity and low immune response remain barriers to effective treatment with immune checkpoint inhibitors [21–23]. The immune microenvironment plays a vital role in cancer development [24]. It is therefore necessary to explore the immune status and immunotherapy response for target indicators; this may aid in regulation of the immune microenvironment and improve the efficacy of immunotherapy in glioma. In this study, we found that FDX1 plays an important role in the migration of glioma cells and invasion of the immune microenvironment; this could be become a new prognostic and immune-related biomarker.

Copper is an essential cellular element for neuronal and immune functions. However, excessive intracellular copper is related to many diseases including cancers [25, 26]. A recent study revealed a novel type of copper-dependent regulated cell death (cuproptosis) which requires mitochondrial respiration. The authors demonstrated that copper-induced cell death occurs via binding of copper to lipoylated proteins of the tricarboxylic acid cycle; this results in a fatty acylated protein-related toxic stress response and loss of iron–sulfur (Fe-S) cluster proteins, eventually leading to cell death [27]. Cuproptosis may play a prominent role in future therapeutic regimens for cancer. Recent studies suggest that cuproptosis may regulate cancer cell death, help overcome the resistance of tumor cells to chemotherapy, and help eliminate defective cells [28, 29]. *FDX1* has been identified as a crucial gene for copper-induced cell death using CRIPSR/Cas9 genome-wide screening; it is an upstream regulator of protein lipoylation. Ferredoxins are ubiquitous proteins with electron transfer activity that act as electron donors for catalytic reactions mediated by mitochondrial cytochrome P450 enzymes [27]. Recent studies suggest that FDX1 affects the progression of multiple human tumors and is involved in many metabolic processes [30]. Zhang et al. found that FDX1 was significantly reduced in hepatocellular carcinoma and that high levels of FDX1 expression correlated with poor prognosis [31]. Evidence suggests that knockdown of FDX1 affects glucose metabolism and fatty acid oxidation in lung adenocarcinomas [32]. There is also considerable evidence that metabolism plays a vital role in the occurrence and progression of human tumors and that aberrant metabolism is closely associated with the immune microenvironment during cancer development [33, 34]. Successful immunotherapy strategies have provided novel opportunities for the treatment of gliomas; this may potentially improve outcomes in these patients. However, the complex immune microenvironment is a major obstacle to immunotherapy in gliomas [35]. Although FDX1 may play a pivotal position in immunotherapy for many cancers, reports regarding its function in gliomas are limited. Studies on the expression of cuproptosis-related genes may improve understanding on immune infiltration; these genes may act as potential predictors and provide targets for intervention in glioma immunotherapy.

In this study, survival analysis showed that upregulation of *FDX1* expression is predictive of a poor prognosis in glioma. The levels of FDX1 expression correlated positively with tumor recurrence, advanced grade, wild type IDH, 1p19q non-codeletion, and chemotherapy status. Stratified analysis based on different clinical parameters revealed that the OS rate was shorter in the high FDX1 expression group than in the low expression group. The potential biological differences between the high- and low-FDX1 expression groups were also analyzed. The findings suggested that the DEGs were involved in the formation of immunoglobulin complexes, blood microparticles, and synaptic membranes (among the cellular components). The DEGs were enriched in defense responses to bacteria, humoral immune responses, and immunoglobulin-mediated immune responses. The above results indicate that FDX1 correlates significantly with several important immunomodulatory functions.

Another important finding was that FDX1 was closely related to the immune status, immune checkpoints, and immunotherapy responses. As one of the most promising strategies to cancer therapy, immunotherapy has shown encouraging success rates in many cancers. However, the current prospects of immunotherapy for glioma are not optimistic. The tumor immune microenvironment plays an indispensable role in immunotherapy for cancer [36, 37]. Our results confirmed that FDX1 was positively associated with relative abundance of most infiltrating immune cells. Additionally, the results demonstrated that human leukocyte antigens, inflammation-promotion, and antigen presenting cell co-inhibition were strong related with FDX1 expression levels. Macrophage (M0, M1, and M2), activated CD4 memory T cell, CD8 T cell, and gamma delta T cell counts were also observed to be elevated in the high-FDX1 expression group. However, monocytes, activated NK cells, and resting CD4 memory T cells showed an opposite trend. The expression of immune checkpoints was also found to be higher in the high-FDX1 expression group than in the low expression group; this further suggests that FDX1 may be a potential target for cancer immunotherapy, as the immune response is often used as a target for cancer therapy.

Our study had some limitations. First, differential expression analyses were performed using a public dataset and some clinical samples were included. However, as the dataset included data from TCGA and CGGA (a database for Chinese glioma patients), the results were accurate. Second, we performed the experiments *in vitro* and animal experiments were required. Finally, immune regulation is a complex process and more detailed mechanisms need to be explored.

This study assessed the correlation between FDX1 expression and immunotherapy response. Patients with glioma having high- and low-FDX1 expression showed differential immunotherapy response. These results suggest that FDX1 plays a pivotal role in cancer immunotherapy. The regulation of FDX1 in glioma cells was also studied; *in vitro* experiments confirmed that the invasive and metastatic abilities of the glioma cells were apparently suppressed after FDX1 knockdown. In this context, the NOD-like receptor signaling pathway plays an important role in the innate immune response. FDX1 silencing-induced inhibitions of cell invasion and migration were found to be associated with inactivation of the NOD-like receptor signaling pathway in glioma. Our findings further confirmed that FDX1 may promote the progression of gliomas by regulating PD-L1 expression via NOD-like receptor signaling pathway activation.

## Supplementary Material

Supplementary Figures

Supplementary Table 1
