# Supplementary material for: FDX1 serves as a prognostic biomarker and promotes glioma progression by regulating the immune response
Source: Aging (Albany NY). 2023 Jun 10;15(11):4963–85. doi: 10.18632/aging.204772 (PMC10292899; doi:10.18632/aging.204772)
Supplement: Supplementary Figures [file aging-15-204772-s001.pdf]

SUPPLEMENTARY FIGURES

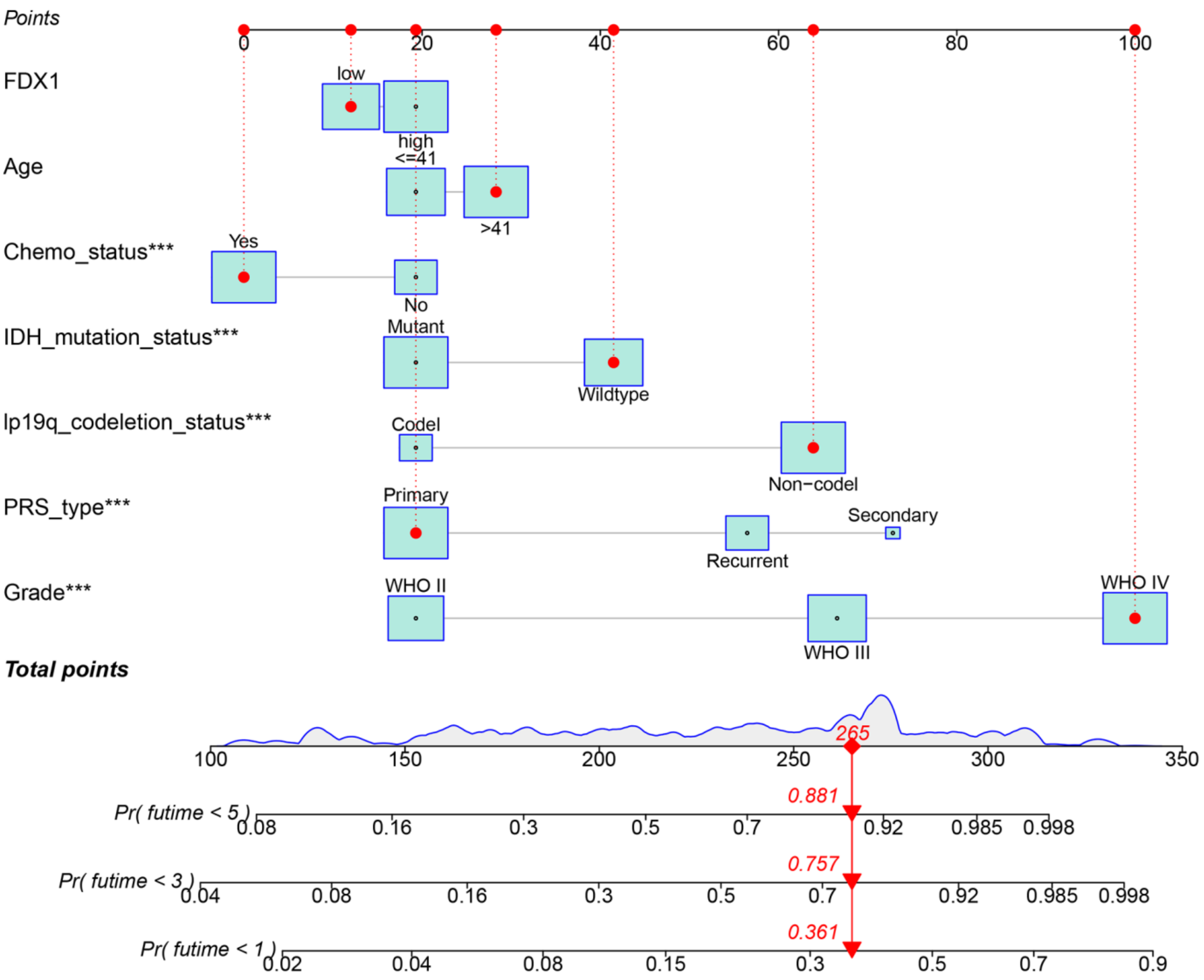

Supplementary Figure 1. Nomogram of FDX1 and clinical parameters for predicting prognosis of glioma patients.

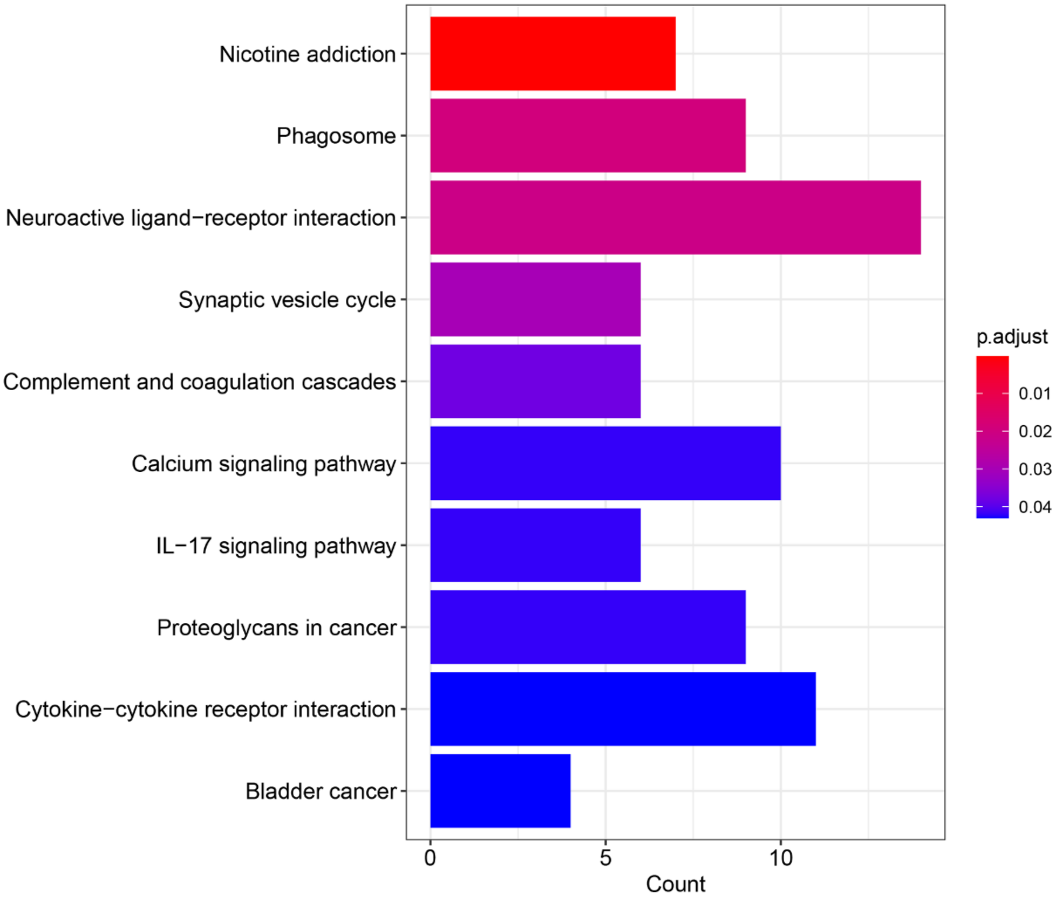

Supplementary Figure 2. KEGG pathway analysis for differentially expressed genes between high- and low-FDX1 expression groups.

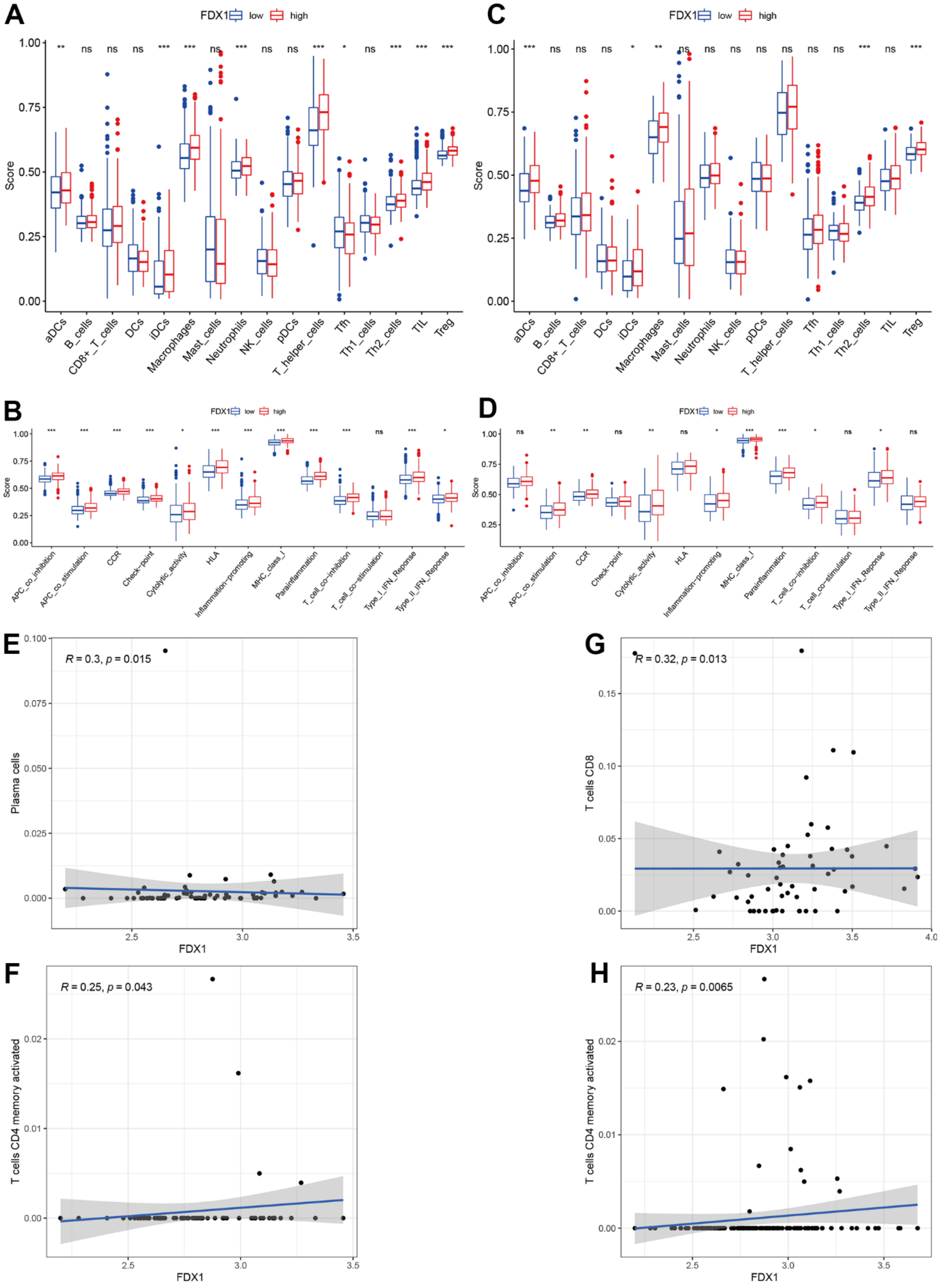

Supplementary Figure 3. Immune status comparison among different tumor types (**A**, **B**) Comparisons of immune status in LGG. (**C**, **D**) Comparisons of immune status in GBM. (**E**, **F**) Correlation of FDX1 with plasma cells and T cells CD4 memory activated in LGG. (**G**, **H**) Correlation of FDX1 with T cells and T cells CD4 memory activated in GBM.

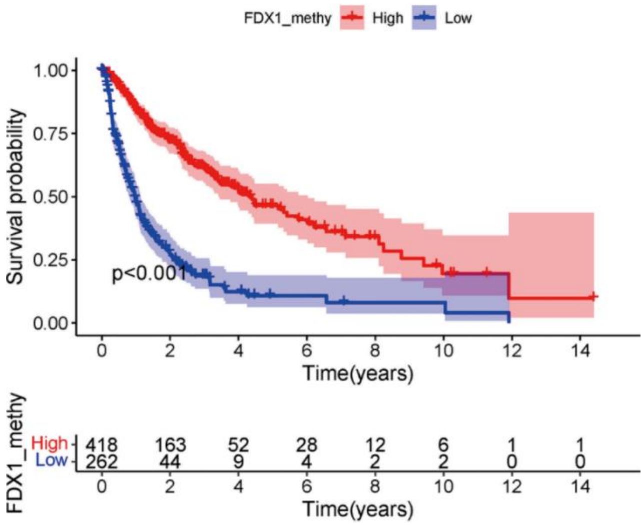

Supplementary Figure 4. Correlation of FDX1 methylation level with clinical prognosis.
