# Supplementary material for: FDX1 serves as a prognostic biomarker and promotes glioma progression by regulating the immune response
Source: Aging (Albany NY). 2023 Jun 10;15(11):4963–85. doi: 10.18632/aging.204772 (PMC10292899; doi:10.18632/aging.204772)
Supplement: Supplementary Table 1 [file aging-15-204772-s002.pdf]

SUPPLEMENTARY TABLE

Supplementary Table 1. Primer sequences for NOD1, PDL1, SOX2, MMP9, and vimentin.

Gene name		Sequence (5` – 3`)
NOD1	forward	CGGATCCATGGAAGAGCAGGGCCACAGTG
	reverse	CCGCTCGAGTCAGAAACAGATAATCCGCTTC
PDL1	forward	AGAAGGAAAGGCAAACAACGAAGAGTC
	reverse	GGAGCCTCGGGAAGCTGCGCAGAACTG
SOX2	forward	AGGATAAGTACACGCTGCCC
	reverse	AACTGTCCATGCGCTGGTT
	reverse	CAGAGGTGTGAGGATGGTGC
MMP9	forward	CGACGTCTTCCAGTACCGAG
	reverse	TTGTATCCGGCAAACTGGCT
Vimentin	forward	GACGCCATCAACACCGAGTT
	reverse	CTTTGTCGTTGGTTAGCTGGT
	reverse	GCACATTGCTCAGTTCATACACC
Vector	forward	ACAACTTTGGTATCGTGGAAGG
	reverse	GCCATCACGCCACAGTTTC
